# Mitochondrial complex I density is associated with IQ and cognition in cognitively healthy adults: an in vivo [^18^F]BCPP-EF PET study

**DOI:** 10.1186/s13550-024-01099-1

**Published:** 2024-04-17

**Authors:** Ekaterina Shatalina, Thomas S. Whitehurst, Ellis Chika Onwordi, Barnabas J. Gilbert, Gaia Rizzo, Alex Whittington, Ayla Mansur, Hideo Tsukada, Tiago Reis Marques, Sridhar Natesan, Eugenii A. Rabiner, Matthew B. Wall, Oliver D. Howes

**Affiliations:** 1 Psychiatric Imaging Group, MRC London Institute of Medical Sciences, Hammersmith Hospital, Imperial College London, London, UK; 2 Institute of Psychiatry, Psychology and Neuroscience (IoPPN), Kings College London, London, UK; 3 Centre for Psychiatry and Mental Health, Wolfson Institute of Population Health, Queen Mary University of London, London, UK; 4 Faculty of Medicine, Imperial College London, London, UK; 5 Invicro, London, UK; 6 Hamamatsu Photonics K.K, Hamamatsu, Japan; 7 Clinical Psychopharmacology Unit, University College London, London, UK

**Keywords:** Mitochondrial complex I (MC-I), General intelligence, [^18^F]BCPP-EF, IQ, Auditory verbal learning

## Abstract

**Background:**

Mitochondrial function plays a key role in regulating neurotransmission and may contribute to general intelligence. Mitochondrial complex I (MC-I) is the largest enzyme of the respiratory chain. Recently, it has become possible to measure MC-I distribution in vivo, using a novel positron emission tomography tracer [^18^F]BCPP-EF, thus, we set out to investigate the association between MC-I distribution and measures of cognitive function in the living healthy brain.

**Results:**

Analyses were performed in a voxel-wise manner and identified significant associations between [^18^F]BCPP-EF DVR_CS−1_ in the precentral gyrus and parietal lobes and WAIS-IV predicted IQ, WAIS-IV arithmetic and WAIS-IV symbol-digit substitution scores (voxel-wise Pearson’s correlation coefficients transformed to Z-scores, thresholded at Z = 2.3 family-wise cluster correction at *p* < 0.05, *n* = 16). Arithmetic scores were associated with middle frontal and post-central gyri tracer uptake, symbol-digit substitution scores were associated with precentral gyrus tracer uptake. RAVLT recognition scores were associated with [^18^F]BCPP-EF DVR_CS−1_ in the middle frontal gyrus, post-central gyrus, occipital and parietal regions (*n* = 20).

**Conclusions:**

Taken together, our findings support the theory that mitochondrial function may contribute to general intelligence and indicate that interindividual differences in MC-I should be a key consideration for research into mitochondrial dysfunction in conditions with cognitive impairment.

**Supplementary Information:**

The online version contains supplementary material available at 10.1186/s13550-024-01099-1.

## Introduction

Mitochondria play a key role in regulating neuronal membrane excitability and provide the majority of the adenosine triphosphate (ATP) necessary for normal brain function [1, 2]. Over recent years, there has been increased discussion of mitochondrial function as a contributing factor to general intelligence [3–5]. This is an attractive hypothesis, as mitochondrial energy production contributes to the maintenance of synaptic signalling, and to whole brain connectivity [3, 6], and thus could contribute to cognitive performance through these and other mechanisms [7, 8].

Modelling mitochondrial dysfunction in mice has been shown to result in cognitive impairment [9]. Mitochondrial dysfunction has also been implicated in many neurological conditions associated with cognitive dysfunction [10–21]. In the human neocortex, reactive oxygen species formation, which is a marker of respiratory chain function, has been linked with intelligence quotient [22]. Thus, one pharmacological approach that attempts to compensate for the energy deficit associated with mitochondrial dysfunction is by targeting the respiratory chain and ATP production [23–25]. Mitochondrial complex one (MC-I) is the largest enzyme complex of the respiratory chain, catalysing the rate-limiting step for oxidative phosphorylation, namely the oxidation of the nicotinamide adenine dinucleotide (NADH [26]).

MC-I function can be manipulated pharmacologically using methylene blue, which targets the electron transport chain and can improve performance on cognitive tasks in humans [27]. At low concentrations, methylene blue enters neural mitochondria to form a renewable redox complex that reduces the production of reactive oxygen species and prevents the pathological blockage of MC-I [27, 28]. In rodent models of Alzheimer’s disease, it has been shown to prevent chronic memory impairment and increase cingulo-thalamo-hippocampal connectivity [6, 29]. In healthy subjects, methylene blue has been shown to increase neural activity measured by functional magnetic resonance imaging (fMRI)  during short-term memory and sustained attention tasks and improve cognitive performance [30].

A new PET probe, 18F-2-tert-butyl-4-chloro-5-{6-[2-(2-fluoroethoxy)-ethoxy]-pyridin-3-ylmethoxy-2 H-pyridazin-3-one ([^18^F]BCPP-EF), binds specifically and with low nanomolar affinity to MC-I, making it possible to investigate MC-I in the living brain [31, 32]. Recent studies in mild cognitive impairment and Alzheimer’s disease indicate that [^18^F]BCPP-EF uptake negatively correlates with cognitive decline in the disease [33, 34] and raises questions regarding how MC-I distribution may relate to cognitive function. Given the evidence discussed above, we set out to test the hypothesis that MC-I distribution is directly related to cognitive function in healthy adults.

## Methods

This study was approved by the London-West London and GTAC ethics committee (Integrated Research Application System reference: 209761, study reference 16/LO/1941) and the Administration of Radioactive Substances Advisory Committee (ARSAC, UK). All methods were carried out in accordance with the updated Declaration of Helsinki (2013). All participants received a description of the study prior to providing written informed consent to participate. 20 healthy participants were recruited (3 F, 17 M, mean age 37 years, age range 20–59 years old). Exclusion criteria included: history of or current substance use disorder (other than tobacco), history of head injury or neurological abnormality, use of any psychoactive medications, significant physical, psychiatric or neurological comorbidity (with the exception of minor self-limiting illnesses), and contraindications to PET or MRI scanning. All subjects had adequate command of English, underwent a structural MRI scan and dynamic PET scan with [^18^F]BCPP-EF and a battery of cognitive tests including the Rey’s Auditory Verbal Learning Test (RAVLT), an abbreviated version of the Wechsler Adult Intelligence scale (WAIS-IV), Trail Making A Test (TMAT) and National Adult Reading Test (NART).

### Positron emission tomography

[^18^F]BCPP-EF was synthesized as previously described [31]. Injected dose information is summarized in Supplemental Table 1. [^18^F]BCPP-EF PET scans were acquired on a Hi-Rez Biograph 6 PET/CT scanner (Siemens, Erlangen, Germany) at the Invicro Clinical Imaging Centre, London. A low-dose CT scan (30 mAs, 130 keV, 0.55 pitch) was performed immediately before each PET scan to estimate attenuation. The tracer was administered as a bolus (20 mL over 20 s) at the start of the scan through a cannula inserted into a cubital or forearm vein. A second cannula was inserted into the radial artery to enable arterial blood collection. Dynamic emission data were acquired over 90 min following radiotracer administration and were reconstructed using discrete inverse Fourier transform reconstruction into 26 frames (frame durations: 8 × 15s, 3 × 60s, 5 × 120s, 5 × 300s, and 5 × 600s). Data were corrected for attenuation, randoms, and scatter.

For the first 15 min of the scan whole-blood activity was measured using a continuous automatic blood sampling system (Allogg AB) at a rate of 5 mL/min. Total blood and plasma radioactivity concentration was also measured (Perkin Elmer 1470 10-well g-counter) from blood sampled at 10, 15, 20, 25, 30, 40, 50, 60, 70, 80, and 90 min after the start of the scan.

### Magnetic resonance imaging data acquisition

T1-weighted magnetisation-prepared rapid acquisition gradient echo (MPRAGE) images were for used for co-registering the PET images. They were acquired on a Siemens Magnetom Prisma 3T scanner (Siemens, Erlangen, Germany) according to the following parameters: repetition time = 2300.0 ms, echo time = 2.28 ms, flip angle = 9°, field of view (FOV) = 256 × 256 mm, 176 sagittal slices of 1-mm thickness, distance factor = 50%, voxel size = 1.0 × 1.0 × 1.0 mm.

### Image analysis and processing

All image data were analysed using the Invicro in-house PET data quantification tool, MIAKAT (version 4.3.7), which implements MATLAB (version R2018b; MathWorks Inc.) and FSL (version 5.0.11; FMRIB) functions for brain extraction and SPM12 (Wellcome Trust Centre for Neuroimaging) for image segmentation and registration.

Brain extraction, grey matter segmentation and rigid-body co-registration to a standard reference space was performed on each subject’s MR image. PET images were registered to this MR image and corrected for motion using frame-to-frame rigid-body registration. The centrum semiovale (CS) region of interest was generated from the automated anatomical labelling template and non-linearly warped onto each subject’s structural MR image for quantifying tracer uptake in pseudo-MNI space for the CS only. Each subject’s PET data were transformed into standard MNI152 space prior to kinetic modelling.

### Tracer kinetic modellings

All time–activity curves (TAC) were fitted in a voxel-wise manner, except for the CS region, where a TAC was plotted for the whole region in pseudo-MNI space to ensure accurate quantification. The multilinear analysis 1 (MA1) model was used to estimate the total volume of distribution (VT). Outlier voxels were removed by thresholding images at a VT of 55, which assumes values above 55 are of supraphysiological level based on previous work using this tracer (described in supplement, supplementary Table 2) [35].

DVR_CS−1_ parametric maps were calculated as the main outcome measure by dividing VT parametric maps for each subject by the CS VT and subtracting 1 to account for non-specific binding.

### Cognitive tests

Rey’s Auditory Verbal Learning Test (RAVLT) is a reliable psychometric instrument used for assessing aspects of episodic memory [36, 37]. The RAVLT consists of a list of 15 words (List A) read to the participant across five consecutive trials. The list is read by the assessor and the participant is immediately asked to recall as many words as they can remember. This procedure is repeated 5 times consecutively (Trials 1 to 5). After that, a new list (List B) of 15 new words is read to the participant and they are asked to immediately recall the words from List B. After this, the examiner asks the participant to recall the words from the first list (Trial 6). After 30 min the participant is asked to recall the words from the first list again (delayed recall). This is followed by the participant being given a grid containing 50 words which include all the words from list A, list B, and additional words that have not appeared previously; participants are asked to circle the words from list A (recognition). Summary scores derived from the RAVLT scores include: total learning (total words remembered trials 1–5), delayed recall score, and recognition scores.

Wechsler Adult Intelligence Scale (WAIS-IV; shortened version) was used to measure intellectual quotient in a reliable and time-effective manner [38]. The abbreviated version used in this study consists of four subtests: (1) Symbol digit substitution test, (2) Arithmetic, (3) Information, and (4) Block design. The symbol digit substitution component consists of nine digit-symbol pairs, followed by a list of digits, under which the subject is asked to write the corresponding symbol as fast as possible. Participants are given a time limit of 120 s to complete as much of the task as possible. The arithmetic component is designed to test mental manipulation, concentration, attention, and numerical reasoning ability. It consists of a set of arithmetic questions that are read out loud to the participant who was asked to answer. The questions increase in their level of difficulty and following two wrong answers the test is stopped and the number of correct responses is recorded as the final score. The information component includes a set of general knowledge questions read to the participant, following two consecutive incorrect responses the test is stopped and the final score of correct responses is recorded. The block design tests spatial visualization ability and motor skill. The participant uses hand movements to rearrange blocks that have various colour patterns on different sides to match a pattern, correct responses within permitted time limits are counted. Administration and scoring were conducted in accordance with the manual to produce a measure of IQ, the main outcome measure for the WAIS [39].

Trail Making A Test (TMAT) is primarily a test of motor speed and visual attention and has good construct validity for measuring working memory [40]. The subjects are asked to draw a line between consecutive numbers as fast as possible and their completion time was used as the main outcome measure.

The National Adult Reading Test (NART) is a commonly used method for measuring premorbid intelligence. The NART was administered in accordance with instructions [41, 42] and transformed to calculate an alternative measure of IQ to the WAIS-IV.

### Statistical analysis

FSL’s (FMRIB Software Library, v5.0.11) fslmaths was used to concatenate [^18^F]BCPP-EF DVR_CS−1_ parametric mages into a 4D dataset. To limit statistical analyses to grey matter voxels only, the 4D image was masked using a 75% probability grey mask derived from the MNI152 template. The AFNI (Analysis of Functional NeuroImages, v17.2.17) 3dTcorrelate module was used to run voxel-wise Pearson’s correlations between BCPP-EF DVR_CS−1_ and cognitive scores for each test, correlating each voxel’s values (across subjects) with the subject’s test scores. A correlation approach to analysis was chosen as it didn’t presume a cause-effect relationship between the measures. Resultant *r*-coefficient images were transformed to *t*-statistical images using fslmaths, using formula 1 below, which were then transformed to *Z*-scores using FSL’s ‘ttoz’ function. FSL’s ‘easythresh’ function was then used to threshold the images at *Z* = 2.3, with cluster extent brain thresholding applied at *p* < 0.05 to correct for multiple comparisons across the brain. Final figures were made by creating a binary mask of significant clusters for each analysis and using this to mask the original *r*-images to show the *r*-coefficients corresponding to the significant findings. In addition to primary measures, for subjects that had data for all cognitive tests, an exploratory composite measure was derived. This was done by transforming scores into *Z*-scores that were averaged across cognitive scales. This measure included RAVLT total words, RAVLT recall, RAVLT recognition, NART, TMAT and the four subscales of the WAIS.

Formula 1:

$$t = \frac{{r\sqrt {n - 2} }}{{\sqrt {1 - {r^2}} }}$$

## Results

**Fig. 1:**
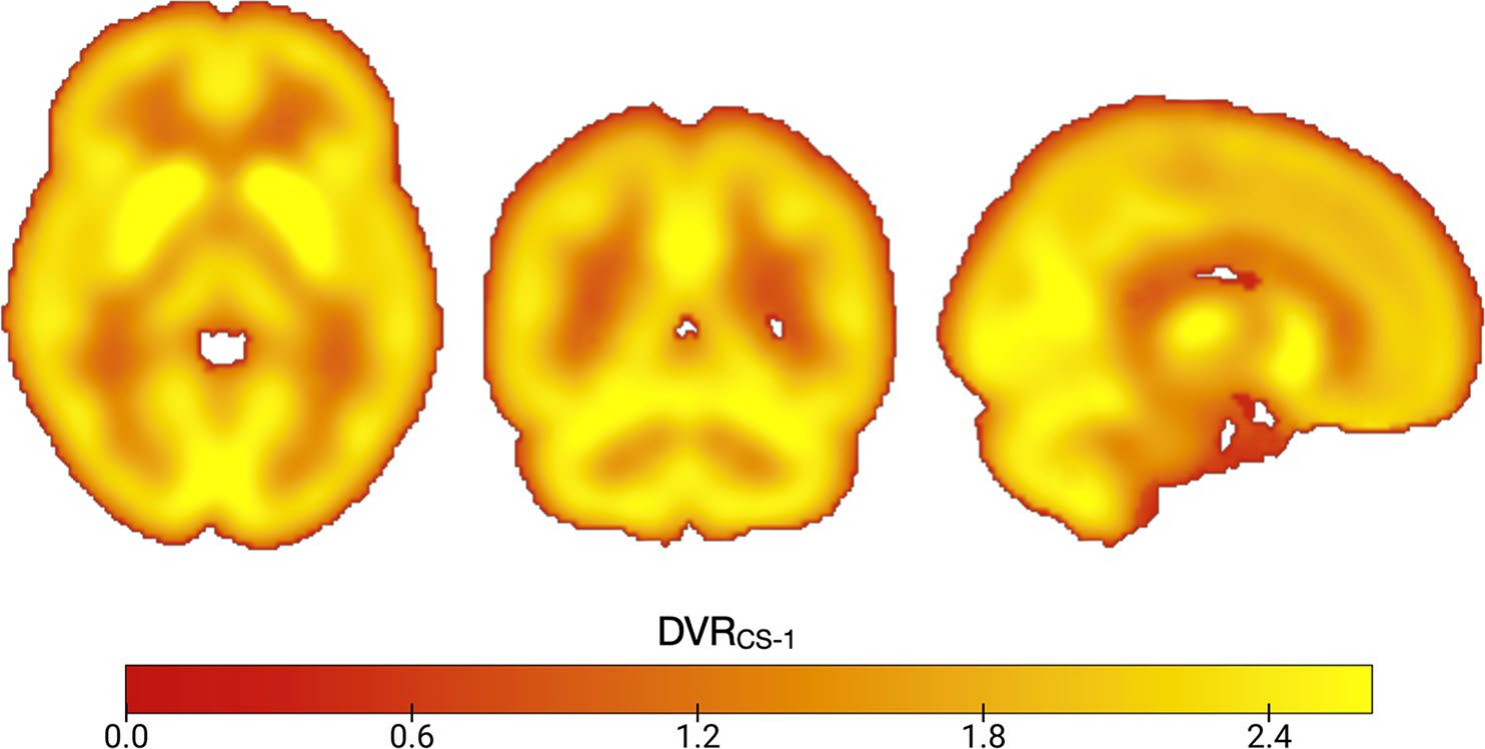
Mean parametric [^18^F]BCPP distribution volume ratio (DVR_CS−1_) images from 20 healthy control subjects, slices shown are x=10.5, y=–52.5, z=–3.2 in MNI152 standard space

All participants completed a [^18^F]BCPP-EF scan, a mean parametric image of [^18^F]BCPP-EF DVR_CS−1_ is shown in Fig. 1. As seen, tracer uptake was greatest in grey matter regions including the cerebellum, occipital lobe and striatum. All participants completed the full RAVLT, except one subject who did not complete the delayed recall and recognition part of the task. 18 participants completed the NART and TMAT. 16 participants completed the WAIS-IV.

**Fig. 2:**
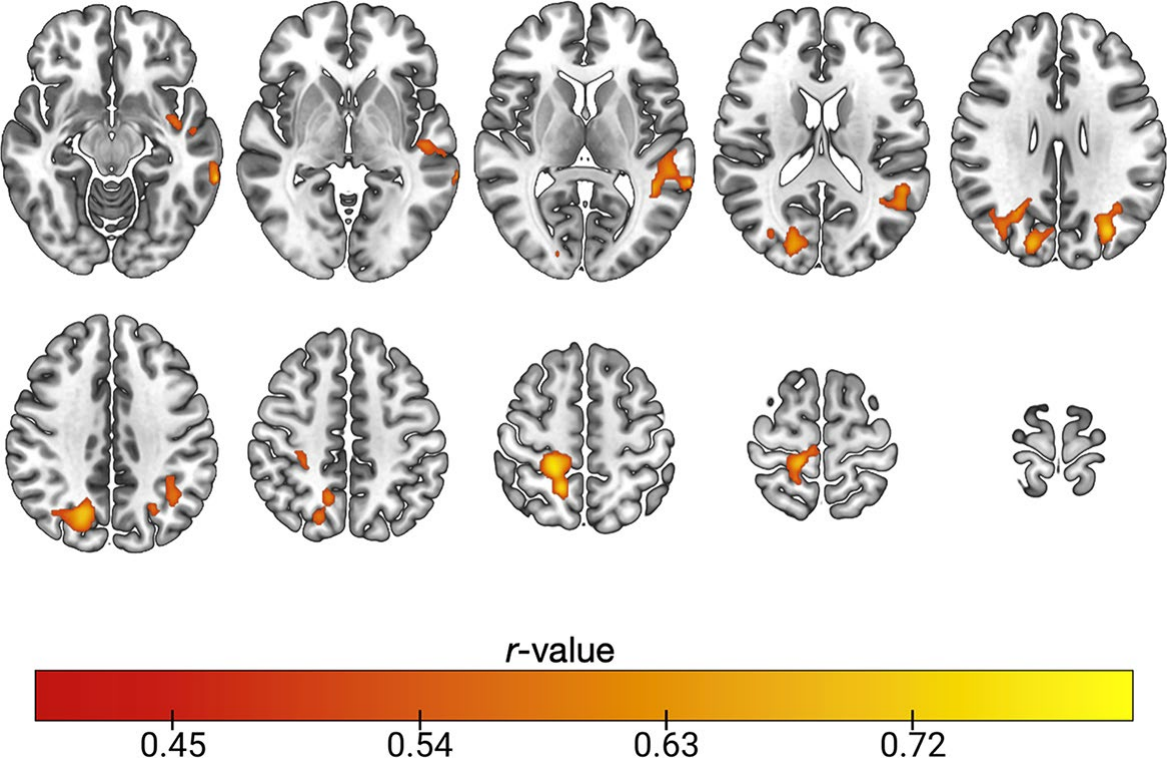
Pearson’s correlation coefficients for clusters where [^18^F]BCPP-EF DVR_CS−1_ was significantly correlated with RAVLT recognition performance in cognitively healthy adults. (*n* = 19, Pearson’s correlation, *Z* > 2.3, cluster threshold *p* < 0.05). Axial slices in MNI152 are: -12 -2 8 18 28; 38 48 56 66 76, results are shown in neurological formal (L = L).

There were no significant associations between [^18^F]BCPP-EF DVR_CS−1_ and IQ as predicted by the national adult reading test, or between BCPP-EF DVR_CS−1_ sand Trail Making A scores in any brain region (*n* = 18, Pearson’s correlation, *Z* < 2.3, cluster threshold *p* > 0.05). There were also no significant associations between RAVLT total learning and delayed recall measures (*n* = 20, Pearson’s correlation, *Z* < 2.3, cluster threshold *p* > 0.05). There were significant associations between RAVLT recognition scores and [^18^F]BCPP-EF DVR_CS−1_ in regions spanning occipital, parietal and temporal areas, including the superior and middle temporal gyrus, postcentral gyrus and precuneus (*n* = 19, Pearson’s correlation, *Z* > 2.3, cluster threshold *p* < 0.05). Correlation coefficients for the significantly correlated clusters are shown in Fig. 2, an unthresholded correlation map is shown in supplementary Fig. 1 for reference.

**Fig. 3:**
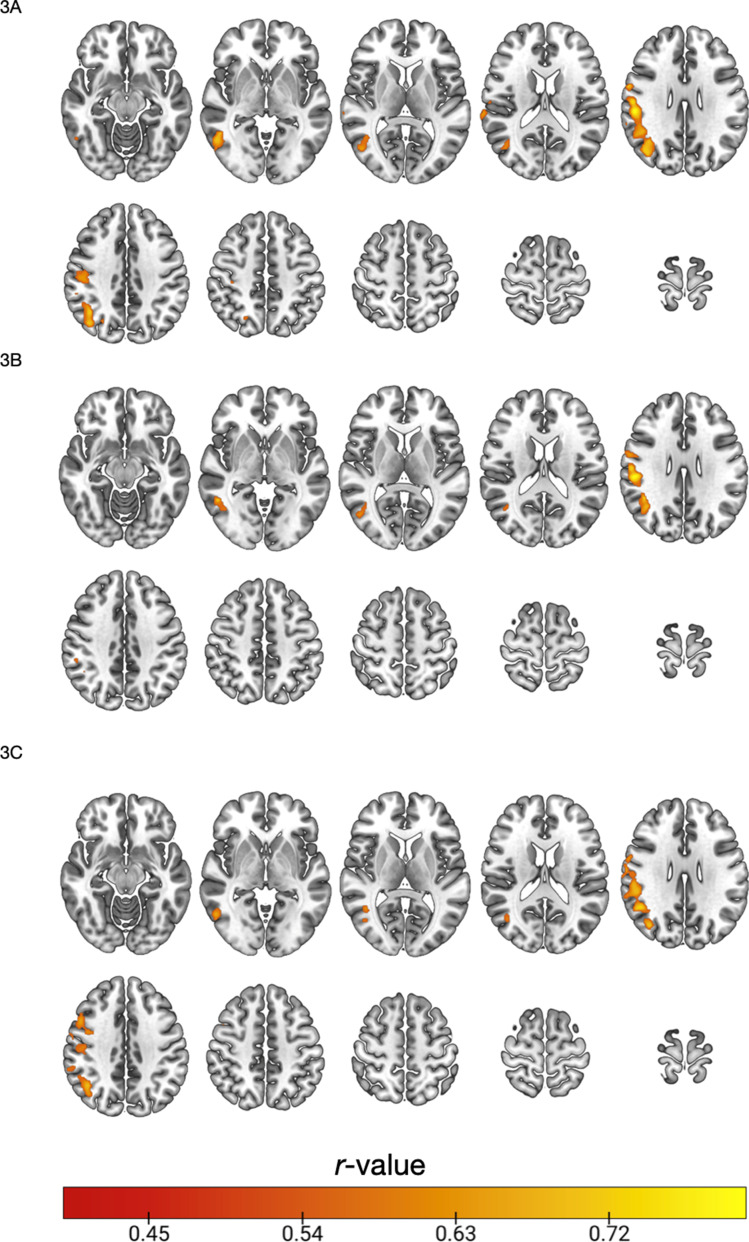
Pearson’s correlation coefficients for clusters where [^18^F]BCPP-EF DVR_CS−1_ was significantly correlated with WAIS-IV predicted IQ (3**A**), WAIS-IV symbol-digit substitution scores (3**B**), WAIS-IV arithmetic scores (3**C**) (*n* = 16, Pearson’s correlation, *Z* > 2.3, cluster threshold *p* < 0.05). Axial slices in MNI152 are: -12 -2 8 18 28; 38 48 56 66 76, results are shown in neurological format (L = L).

There were significant associations between [^18^F]BCPP-EF DVR_CS−1_ distribution and WAIS-IV predicted IQ in regions spanning the precentral gyrus and parietal lobe (Fig. 3A) (*n* = 16, Pearson’s correlation, *Z* > 2.3, cluster threshold *p* < 0.05). Exploratory analyses into what subcomponents of the WAIS contributed to these results showed that there were significant associations between [^18^F]BCPP-EF DVR_CS_ and the WAIS-IV symbol substitution and arithmetic scores, but not between DVR_CS−1_ and scores on the block design and information subcomponents of the WAIS-IV. Figure 3 shows regions where MC-I distribution, as measured by [^18^F]BCPP-EF DVR_CS_, was significantly correlated with WAIS-IV symbol substitution scores (3B), showing regions spanning the precentral gyrus, parietal and occipital regions (*n* = 16, Pearson’s correlation, *Z* > 2.3, cluster threshold *p* < 0.05) and arithmetic scores (3C), showing significant regions spanning the middle frontal gyrus, post-central gyrus and parietal cortex. Unthresholded correlation maps corresponding to Fig. 3 are shown in supplementary Fig. 2 for reference. There were no significant correlations between the composite cognitive measure capturing data from all of the scales used (*n* = 16) and no significant negative associations between [^18^F]BCPP-EF DVR_CS−1_ and any cognitive measures assessed in this study.

## Discussion

Our main finding is the positive association between MC-I density, as measured by [^18^F]BCPP-EF DVR_CS−1_, in the precentral gyrus and parietal lobes of healthy adults and IQ, as predicted by the shortened WAIS-IV. This was likely driven by the arithmetic and symbol digit substitution components of the WAIS-IV which was used to calculate IQ, where scores were associated with [^18^F]BCPP-EF DVR_CS−1_ in parietal and occipital regions for both tasks. Additionally, [^18^F]BCPP-EF DVR_CS−1_ in the middle frontal gyrus and post-central gyrus was associated with arithmetic scores and [^18^F]BCPP-EF DVR_CS−1_ in the precentral gyrus was associated with symbol-digit substitution performance. We also found that RAVLT recognition was associated with [^18^F]BCPP-EF DVR_CS−1_ in the middle frontal gyrus, post-central gyrus, occipital and parietal regions.

Taken together these findings are in regions where we may expect to see effects of MC-I on brain function. Verbal memory recognition has been tied to activation of the posterior parietal cortex [43]. The parietal lobe, particularly the intra-parietal sulcus is also a key region involved in number and magnitude processing [44], with a recent meta-analysis of mental arithmetic fMRI studies identifying parietal regions similar to those we show in this study [45]. As well as this, the frontoparietal network, which spans the dorsolateral prefrontal and parietal cortices is reliably activated during executive function tasks and would underlie the effective completion of the tasks we used in this study [46]. Thus, our findings raise the question of whether increased MC-I density may contribute to better cognitive performance by increasing the overall energy availability within certain brain networks involved in cognitive tasks. Mitochondrial DNA measured in post-mortem human brain samples can also be interpreted as an indicator of mitochondrial availability. It is present in the highest levels in the parietal cortex in the healthy brain and is significantly lower in this region in individuals with Alzheimer’s disease compared to those with no cognitive impairment [47]. Magnetic resonance spectroscopy data also suggests that subtle changes in episodic memory performance in elderly subjects may be linked to increased mitochondrial energy capacity in the precuneus [48], which is a region known to be sensitive to reductions in glucose consumption [49] and exhibits some of the earliest signs of metabolic deficit in Alzheimer’s disease [50]. [^18^F]FDG PET provides further evidence linking metabolic markers with cognitive decline [51]. For example, reduced glucose metabolism in the posterior cingulate cortex is associated with the transition from mild cognitive impairment to Alzheimer’s disease [52], while in Parkinson’s [^18^F]FDG PET measures in posterior temporo-parieto-occipital association areas correlate with cognitive performance [53].

The medial temporal lobe, where we saw a relationship between MC-I and RAVLT recognition scores, is a key region involved in recognition memory [54]. In frontotemporal dementia, a patient’s education level has also been found to predict existing [^18^F]FDG PET hypometabolism in the left temporal lobe where patients had the same cognitive performance [55]. This, together with our study suggests that individual differences in MC-I function and metabolic functional reserve may contribute to variance in disease and are a key consideration for studies investigating this. Mitochondrial oxidative stress is also associated with temporal lobe epilepsy (TLE) and has been investigated as a contributing factor to memory impairment and cognitive dysfunction in epilepsy, via the cAMP response element binding protein (CREB) and its downstream signalling pathways [56]. However, one study showed that language and memory impairment in drug-resistant temporal lobe epilepsy is not associated with the activities of mitochondrial enzymes Complex I, Complex II, Complex IV or Succinate dehydrogenase [57]. Given that our study assessed global MC-I distribution an important consideration is that in addition to changes in mitochondrial function, differences in tracer uptake may reflect a greater mitochondrial number, both per cell or per synapse or as a function of greater synaptic density.

## Strengths and limitations

[^18^F]BCPP-EF binds to mitochondrial complex one with high affinity (K_I_=2.3), and in competition studies is almost completely displaced by rotenone, which is known to be a highly specific inhibitor of MC-I function [32, 58–60]. This suggests that non-specific binding for [^18^F]BCPP-EF in the mammalian brain is low. We reported distribution volume ratio, which is calculated as the volume of distribution in a region or voxel of interest, divided by the non-displaceable binding, with the latter being comprised of non-specific binding and free tissue concentration of the radioligand [61]. In practice, the value for non-displaceable binding is derived from a region presumed to have no, or very low, specific binding [61]. In our study, we calculated DVR_CS−1_ as the volume of distribution calculated in a particular voxel, divided by the volume of distribution in the centrum semiovale followed by subtracting 1, in line with other recent studies using [^18^F]BCPP-EF [34, 35]. However, there is substantial evidence that mitochondria are present in white matter, albeit to a lesser extent than in grey matter, where a high density of synapses necessitates higher energy production capacity [62]. Since this means there would be some degree of specific binding in the reference region, this may affect the interpretation of DVR_CS−1_ for this tracer, as it is relative to a region with MC-I, and reduces the proportion of specific binding that we were able to quantify in this study [61]. In addition to this, our method of evaluating relationships between MC-I and cognitive measures was a correlation-based approach. While this is useful for this type of exploratory analysis, as it does not assume a cause-effect relationship between the measures it limits the conclusions we are able to draw from our results.

Previous research investigating the link between MC-I distribution and cognition has been carried out in a small sample of Alzheimer’s patients and matched controls [63]. Measures of cognition included the Mini-Mental State Examination [64], the Weschler Memory Scale – Revised [65], the Frontal Assessment Battery [66], Addenbrooke’s Cognitive Examination III [67], the Repeatable Battery for Assessment of Neuropsychological Status (RBANS) [68] and that National Adult Reading Test (NART) [41]. These studies found no associations between cognitive measures and [^18^F]BCPP-EF in any brain region in age-matched controls [33, 34]. However, the cognitive measures used in these studies were designed to distinguish those with disease-associated cognitive impairment from those with normal cognitive functioning and non-disease populations vary relatively little in their scores on such measures [64–66]. Our study used measures designed to demonstrate inter-individual differences in cognitive functioning and intelligence in healthy individuals, and as such, is better designed to demonstrate correlations between [^18^F]BCPP-EF and cognitive function in a sample of healthy controls.

Our study only had one measure in common with one of these previous studies [33], the NART, and we replicated their finding by not demonstrating any correlation between scores on that test and [^18^F]BCPP-EF DVR_CS−1_. The NART is a test designed to measure premorbid intelligence in dementia, as knowledge of vocabulary is thought to be relatively preserved in the disease [69]. As such, it is a proxy measure of intelligence during the later stages of neurodevelopment, correlates moderately with IQ at age 11 (*r* = 0.6), and is not a preferred measure for current intelligence [69]. Furthermore, as a measure of crystallized intelligence, it might not rely on high-energy networks involved in attentional control or executive function [70, 71], unlike tasks like arithmetic and digit-symbol coding which directly engage these systems.

One final limitation of this study is that a sample of 16–20 participants is modest in the context of a cognitive neuroscience study. While this is common within PET literature, our sample lacked the power to detect weak associations across the cognitive measures. Thus, the absence of significant relationships does not preclude a weaker relationship, although the clinical significance of weak associations is questionable.

### Implications for understanding of intelligence

Our study shows that scores on a validated measure of general intelligence correlate with uptake of [^18^F]BCPP-EF in regions including the precentral gyrus and parietal lobe. Variation in [^18^F]BCPP-EF may be due to variation in MC-I protein concentrations, secondary to genetic variation in genes coding for one of the 45 MC-I subunits [72], variation in one of the many more genes associated with transport assembly or maintenance of MC-I, or degradation of MC-I by reactive oxygen species [73]. Alternatively, variation in [^18^F]BCPP-EF may be related to overall mitochondrial number or may be reflective of variation in synaptic density, which has also been shown to be altered in numerous disease processes associated with cognitive decline [74–77].

This study lends some support to the theory that individual differences in mitochondrial functioning may underlie individual differences in intelligence [78]. This theory grew out of observed correlations between measurements of a wide variety of perceptual, sensory and cognitive processes, termed the “positive manifold” [79]. Such measurements undertaken at age 11 were later shown to be correlated with a large number of health outcomes in later life, with higher scores on a range of psychometric testing increasing the odds of surviving past 79 years [80]. Similarly, the decline in psychometric scores that occurs with age correlates with the decline in function of other organ systems, with the correlations observed most strongly between organ systems with higher metabolic demand [81]. There is an abundance of evidence that mitochondrial respiratory function declines with age, that perturbations of mitochondrial function can accelerate the ageing process, that mitochondrial DNA mutations accumulate with age throughout the body, and that measures of mitochondrial function correlate with cognitive decline during the ageing process (reviewed in [81, 82]). Thus, this study raises the question of whether the decline in mitochondrial function with age could underlie decline in cognitive function with age.

The relationship between mitochondrial function and cognition has been largely investigated in the context of either neurological disorders or mitochondrial disorders that are associated with global cognitive dysfunction, as well as focal deficits in cognitive functions [83, 84]. Lactate is produced by anaerobic respiration, and levels of lactate increase when oxidative phosphorylation in mitochondria is impaired [85]. Levels of lactate in the cerebral spinal fluid correlate with the degree of cognitive impairment in patients with Mitochondrial encephalomyopathy, lactic acidosis, and stroke-like episodes (MELAS) [85]. This indicates that the degree of mitochondrial impairment, albeit measured indirectly, correlates positively with the degree of neurocognitive impairment in mitochondrial diseases. Similarly, some have claimed that evidence of cognitive dysfunction in obesity (reviewed in [86]), combined with evidence of mitochondrial dysfunction supports a relationship between variation in mitochondrial function, but we are not aware of direct reporting of correlations between mitochondrial dysfunction and cognition in the condition [78, 86]. Recent evidence from humans suggests that uptake of [^18^F]BCPP-EF correlated positively with cognitive performance in Alzheimer’s disease and mild cognitive impairment [33, 34]. However, we are not aware of other in vivo studies demonstrating a relationship between brain mitochondrial complex one levels and individual differences in measures of current general intelligence cognitively healthy adults. Further research in larger cohorts is required to validate our findings, including in samples with a greater proportion of female participants, across a wider range of normal cognitive ability and wider age range. Pharmacological studies using methylene blue and functional magnetic resonance imaging or electroencephalography would also serve as a useful tool to investigate the mechanism through which improving mitochondrial function may improve cognition at the whole-brain level.

## Conclusions

MC-I density in the parietal lobe and other regions, as measured with [^18^F]BCPP-EF DVR_CS−1_, is associated with IQ, mental arithmetic, symbol-digit substitution and verbal recognition scores in cognitively healthy adults. This lends some support to the theory that mitochondrial function may contribute to general intelligence and has implications for studies investigating the role of mitochondrial dysfunction in cognitive impairment.

### Electronic supplementary material

Below is the link to the electronic supplementary material.

Supplementary Material 1

## Data Availability

The datasets used and/or analysed during the current study are available from the corresponding author on reasonable request.
