# Supplementary material for: Mitochondrial complex I density is associated with IQ and cognition in cognitively healthy adults: an in vivo [18F]BCPP-EF PET study
Source: EJNMMI Res. 2024 Apr 17;14:41. doi: 10.1186/s13550-024-01099-1 (PMC11024075; doi:10.1186/s13550-024-01099-1)
Supplement: Supplementary file 1 — Supplementary Material 1 [file 13550_2024_1099_MOESM1_ESM.docx]

**Supplementary materials:**

Supplemental table 1: Demographic details and [^18^F]BCPP-EF injected mass, activity and specific activity.

| Subject number | Age | Sex | Weight in Kg | Injected Activity (BCCP) | Mass (BCPP) | Specific activity (BCPP) |
| --- | --- | --- | --- | --- | --- | --- |
| 001 | 53 | M | 85.9 | 97.36 | 0.12 | 334.714 |
| 002 | 25 | M | 69.3 | 91.26 | 0.05 | 718.032 |
| 003 | 27 | M | 78.9 | 92.31 | 0.04 | 903.168 |
| 004 | 59 | M | 78.1 | 86.75 | 0.1 | 339.572 |
| 005 | 52 | M | 82 | 91.45 | 0.07 | 527.67 |
| 006 | 42 | M | 93.6 | 88.54 | 0.11 | 335.04 |
| 007 | 53 | M | 102 | 86.57 | 0.04 | 846.974 |
| 008 | 39 | M | 113.3 | 90.6 | 0.06 | 639.459 |
| 009 | 49 | M | 93.2 | 96.17 | 0.23 | 170.279 |
| 010 | 34 | M | 112 | 91.14 | 0.14 | 264.288 |
| 011 | 50 | M | 93.8 | 96.57 | 0.08 | 477.999 |
| 012 | 25 | M | 106.3 | 93.52 | 0.15 | 253.389 |
| 013 | 30 | M | 70.2 | 83.57 | 0.08 | 426.51 |
| 014 | 38 | M | 98.8 | 90.15 | 0.16 | 227.096 |
| 015 | 21 | F | 63.7 | 92.56 | 0.12 | 319.752 |
| 016 | 29 | M | 69.7 | 94.09 | 0.05 | 99.34 |
| 017 | 20 | M | 69.8 | 92.21 | 0.11 | 325.192 |
| 018 | 21 | F | 67.1 | 91.92 | 0.18 | 203.544 |
| 019 | 24 | M | 86.9 | 94.03 | 0.25 | 152.622 |
| 020 | 29 | F | 68.8 | 83.93 | 0.16 | 213.182 |

**Calculation of supraphysiological threshold for [^18^F]BCPP-EF**

Outlier voxels are an issue when applying the MA1 model in a voxelwise manner. To calculate a threshold for removing outlier voxels we used mean volume of distribution (VT) values previously published for [^18^F]BCPP-EF for a range of brain regions (Mansur, Rabiner et al. 2020) (see supplementary table 2 below), followed by calculating the standard deviation and calculating a mean±3SD VT value for each region, which was assumed as a supraphysiological threshold. The highest uptake for the tracer is in the putamen, where the mean±3SD VT value was 53.38. This was rounded to 55 and used as a threshold for our data to remove outlier voxels.

Supplementary table 2, from Mansur et al., 2020

| **Region** | **Mean VT** | **CoV** | **SD** | **Mean+2SD** | **Mean+3SD** |
| --- | --- | --- | --- | --- | --- |
| Putamen | 34.0 | 0.19 | 6.46 | 46.92 | 53.38 |
| Ventral striatum | 31.6 | 0.2 | 6.32 | 44.24 | 50.56 |
| Cerebellum | 30.6 | 0.16 | 4.896 | 40.392 | 45.288 |
| Parietal lobe | 26.1 | 0.2 | 5.22 | 36.54 | 41.76 |
| Insular cortex | 26.6 | 0.17 | 4.522 | 35.644 | 40.166 |
| Caudate | 20.4 | 0.32 | 6.528 | 33.456 | 39.984 |
| Frontal cortex | 24.8 | 0.2 | 4.96 | 34.72 | 39.68 |
| Temporal lobe | 24.8 | 0.17 | 4.216 | 33.232 | 37.448 |
| Thalamus | 22.9 | 0.2 | 4.58 | 32.06 | 36.64 |
| Hippocampus | 21.7 | 0.18 | 3.906 | 29.512 | 33.418 |
| Substantia Nigra | 20.9 | 0.14 | 2.926 | 26.752 | 29.678 |
| Brain Stem | 17.5 | 0.17 | 2.975 | 23.45 | 26.425 |
| Centrum Semiovale | 11.9 | 0.17 | 2.023 | 15.946 | 17.969 |

Mansur, A., E. A. Rabiner, R. A. Comley, Y. Lewis, L. T. Middleton, M. Huiban, J. Passchier, H. Tsukada and R. N. Gunn (2020). "Characterization of 3 PET tracers for quantification of mitochondrial and synaptic function in healthy human brain: 18F-BCPP-EF, 11C-SA-4503, and 11C-UCB-J." Journal of Nuclear Medicine **61**(1): 96-103.


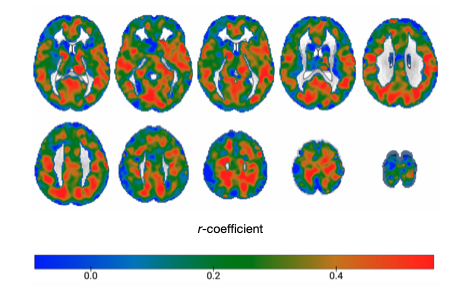


Supplementary figure 1: Pearson’s correlation coefficients for voxel-wise correlations between [^18^F]BCPP-EF DVR_CS-1_ and RAVLT recognition performance in cognitively healthy adults. (*n*=19, Pearson’s correlation, unthresholded). Axial slices in MNI152 are: -12 -2 8 18 28; 38 48 56 66 76, results are shown in neurological formal (L=L)


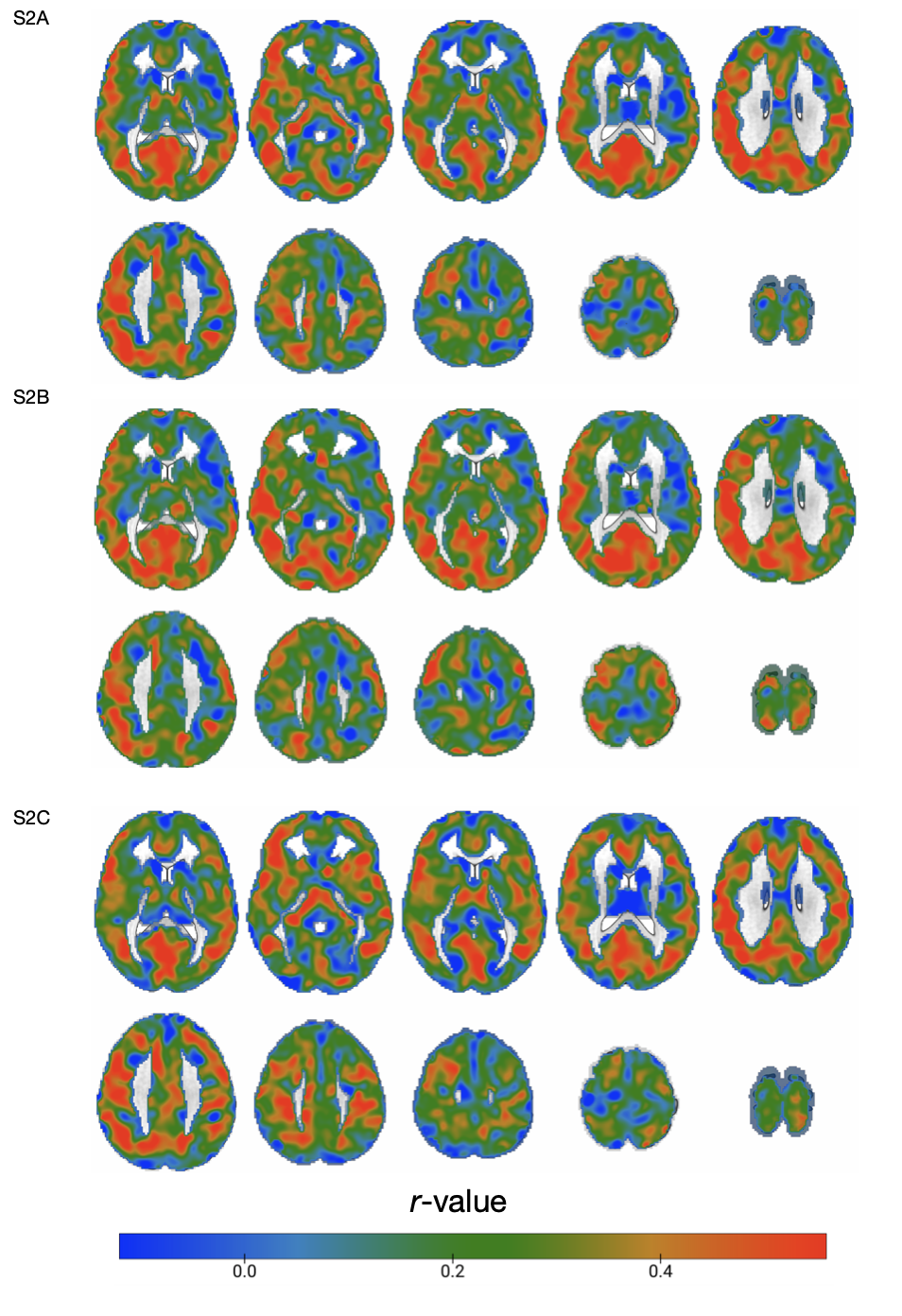


Supplementary figure 2: Pearson’s correlation coefficients for voxel-wise correlations between [^18^F]BCPP-EF DVR_CS-1_ with WAIS-IV predicted IQ (S2A), WAIS-IV symbol-digit substitution scores (S2B), WAIS-IV arithmetic scores (S2C) (*n*=16, Pearson’s correlation, unthresholded). Axial slices in MNI152 are: -12 -2 8 18 28; 38 48 56 66 76, results are shown in neurological format (L=L).
